# Environmentally Sustainable and Green Polymeric Method for Chitosan (CH) Film Synthesis Using Natural Acids and Impact of Zinc Ferrite Nanoparticles (NPs) on Water Solubility (WS) and Physical Properties

**DOI:** 10.3390/polym16243466

**Published:** 2024-12-12

**Authors:** Dilawar Hassan, Ayesha Sani, Ghulam Qadir Chanihoon, Aurora Antonio Pérez, Muhammad Ehsan, Ana Laura Torres Huerta

**Affiliations:** 1 School of Engineering and Sciences, Tecnologico de Monterrey, Atizapan de Zaragoza C.P. 52926, Estado de Mexico, Mexico; a01754344@tec.mx (A.S.); a.antonio@tec.mx (A.A.P.); atorresh@tec.mx (A.L.T.H.); 2 National Centre of Excellence in Analytical Chemistry (NCEAC), University of Sindh, Jamshoro 76080, Pakistan; chemistqadir@gmail.com; 3 Centro de Bachillerato Tecnológico Agropecuario, 162. Carr. Mexico-Veracruz Vía Texcoco km 95, Francisco I. Madero C.P. 90280, Tlaxcala, Mexico; muhammadehsan2000@yahoo.com

**Keywords:** chitosan films, water solubility, sustainable systems, environmental nanotechnology, mechanical properties, green polymers

## Abstract

Currently, there is a rush to develop green polymeric films such as biodegradable chitosan (CH) films to control and prevent plastic pollution from degrading the environment. This study reports a novel and sustainable green approach to the development of CH films using lemon juice (LJ) and lemon peel extract (LPE), the latter to dilute the LJ. The LPE was also utilized for the synthesis of ZnFe_2_O_4_ nanoparticles (NPs), adding to this work’s novelty. The crystalline size of the ZnFe_2_O_4_ NPs was computed to be ~16 nm. The introduction of 1% and 2% ZnFe_2_O_4_ NPs improved not only the mechanical properties of the films, but also their barrier properties and water solubility (WS). The tensile strength increased from 0.641 MPa to 0.835 MPa when 2% NPs were incorporated, which is almost 1.30 times greater; the NPs also enhanced the surface strength by 2.66 times, which was demonstrated by the puncture strength. The introduction of NPs occupied the vacant spaces and improved the barrier capabilities of the CH film by reducing the water vapor permeability (WVP) value from 8.752 ± 0.015 for bare CH films to 6.299 ± 0.009 for 2% NP-containing CH films. Overall, the introduction of ZnFe_2_O_4_ NPs boosted the mechanical and barrier properties of the CH films, and offers a promising method for developing sustainable, eco-friendly, and biodegradable polymeric films for potential packaging and medical applications to contribute to circular economic efforts.

## 1. Introduction

Increasing pollution and growing demand for sustainable materials have attracted extensive research on natural alternatives to surpass conventional and industrially driven components in numerous fields [1]. Since the 1950s, the world has produced 8.3 billion tons of plastic [2], which has been introduced to our land and ecosystem. One area of research interest is the development of green bio-based polymeric films, for their applications ranging from food preservation to use in the medical industry [3]. For the development of bio-based films, various types of polymers are being utilized, including polylactic acid (PLA) [4], polyglycolic acid (PLGA) [5], poly-ε-caprolactone (PCL) [6], and polysaccharides [7].

Polysaccharides are monosaccharide units joined to form long chains by glycosidic bonding [8]. In polysaccharides, cellulose, starch, and chitosan (CH) are most widely used for their biodegradable nature. Starch films are very brittle, with poor mechanical properties [9], while cellulose films have poor barrier properties against gases and moisture [10]. CH, on the other hand, has wide applications and is the best candidate due to having better barrier properties and mechanical strength [11]. Further, CH is known for its biocompatibility, biodegradable, and antimicrobial properties [12].

The dissolution of CH requires the use of industrially driven acids such as acetic acid and citric acid [13]. Citric acid increases the barrier and mechanical properties of the films, but these acids are produced using methods that are unsustainable as they pose energy conservation concerns as well as environmental concerns [14]. Researchers are striving to develop methods that are more sustainable and greener, which utilize less industrially produced chemicals to make film development feasible and environmentally friendly. This has created an opportunity area, which we have employed, by replacing the use of analytical-grade acids with the naturally occurring acid in lemon juice (LJ).

Researchers are also introducing various types of nanomaterials to enhance films in terms of their barrier properties and mechanical strength. There are various methods that could be employed to synthesize nanoparticles (NPs), such as physical, chemical, biological, and green chemical (bio-chemical) methods [15]. The synthesis of NPs using chemical synthesis has various toxic byproducts that make this method disadvantageous [16], while the physical method for NP synthesis requires a pure metal source and high energy, making it non-economical [17]. The biological method is safe and biofriendly, but due to the slow rate of microbial growth, it is time-consuming for NP synthesis [18]. The green chemical method, on the other hand, has many advantages, such as being time-saving and more eco-friendly [19]. Studies have reported that the introduction of ZnO [20], NiO [21], SiO_2_ [22], and Fe_2_O_3_ [23] NPs, among others provides the CH films with improved properties. For instance, W. Dong’s group developed SiO_2_ NP-containing CH films and found that they improved the mechanical strength of the films [22]. The use of NPs also provides films with better UV light barrier properties, as the NPs are known to have narrower band gaps [24]. For instance, ZnO NPs are reported to have a band gap of 3.37 to 3.44 eV [25] and Fe_2_O_3_ NPs reportedly have a 2.77 eV band gap [26]. However, the merging of NPs to develop bimetallic NPs can be performed to obtain a better and narrower band gap; Roumaih, K. et al. found that ZnFe_2_O_4_ NPs had a band gap of 1.77 eV [27].

For the development of NPs, chemical methods are employed [15]. Although chemical synthesis has a high product yield, it is not considered a sustainable method, as it employs analytical-grade chemicals, which are industrially produced and biologically and environmentally toxic [28]. During nucleation, the NPs have reactive surfaces and start accumulating toxic byproducts onto their surfaces to achieve neutrality, hence becoming toxic in themselves too [29]. Furthermore, after NP synthesis, the left-over solutions are also quite toxic in nature; therefore, many researchers are employing the green chemical approach, which is both environmentally friendly and biofriendly [30].

This research, therefore, aims to bridge these gaps through the development of a biopolymer film using CH, where LJ is used as an alternative to citric acid for solubilizing CH, which also provides the novelty of this research. A further novel aspect of this research is based on the 100% use of lemons, as the zinc ferrite NPs were synthesized using lemon peel extract (LPE) in water. These green synthesized NPs were further reinforced in the CH films. The mechanical properties improved by adopting this approach are consequently in agreement with the principles of green chemistry, and, in turn, offer a path towards sustainability in the development of new materials. The reinforced nanomaterials further play their role and provide nanofiller and/or bind with the CH chain to give them modified barrier properties. The results from this study could reveal the potential use in biopolymer films of natural acids and synthesized NPs in a green way, opening a path for their application in areas where performance and sustainability are critical.

## 2. Materials and Methods

Lemons were obtained from the Tianguis Villas de la Hacienda area, State of Mexico, Mexico. Other chemicals and reagents including Glycerol, 1,1—diphenyl-2-picrylhydrazyl, Iron Sulfate heptahydrate (FeSO_4_·7H_2_O), zinc acetate ((CH_3_CO_2_)_2_Zn), and CH (50–190 kDa) were all analytical grade and purchased from Merck, Toluca, Mexico.

### 2.1. Lemon Juice and Lemon Peel Extract

Lemons (2 kg) were initially pre-washed with tap water followed by distilled water to eliminate any surface residues, then allowed to dry at room temperature. The purchased lemons were cut into two halves, using an autoclaved knife, and juice was squeezed into a 1000 mL beaker using a pre-washed juice squeezer. The collected LJ was filtered using Whatman # 40 filter papers to remove any suspended solids and nectar, and was stored in a 1000 mL air-tight bottle at 4 °C for further use. To obtain the LPE and phytochemicals inside the LPE by heating at 60 °C, the peels were heated in DI water with a concentration of 10 g/100 mL, under constant stirring (600 rpm) for 3 h. The resulting solution was filtered thrice using Whatman # 40 filter paper, transferred into an air-tight bottle, and stored at 4 °C until required.

### 2.2. Synthesis of Zinc Ferrite Nanoparticles

**Figure 1:**
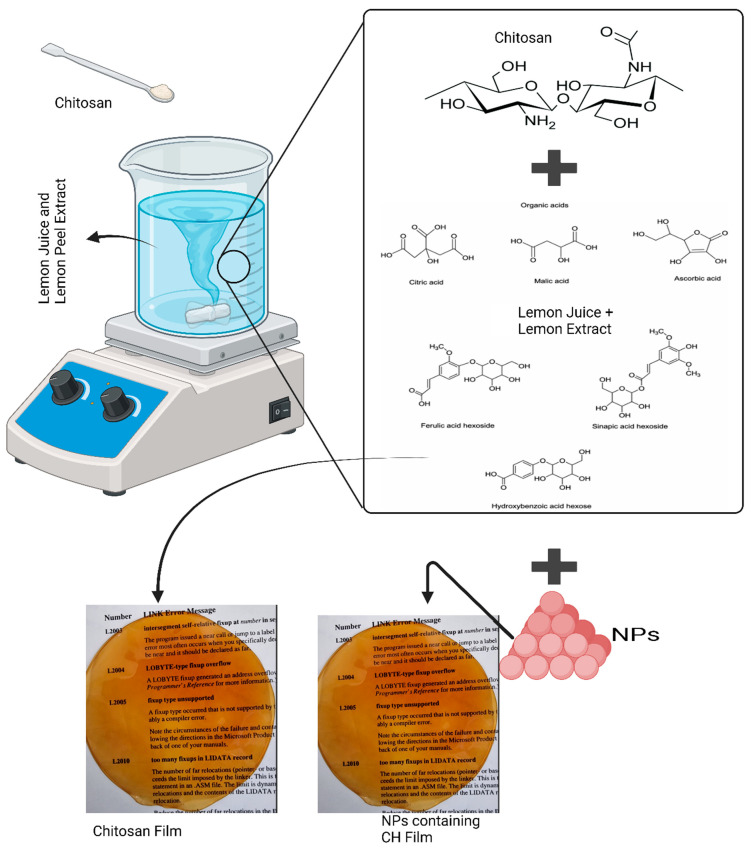
Schematic for chitosan (CH) films and nanoparticle (NP)-containing CH films.

A previously established method was employed for the synthesis of ZnFe_2_O_4_ NPs [19,31]. In brief, a 2:1 molar ratio of salts of iron sulfate and zinc acetate was dissolved in 50 mL of distilled water in a 100 mL beaker, as shown in Figure 1, with stirring at a constant heat of up to 70 °C for 2 h. The salt solution was then transferred to a 250 mL beaker containing 100 mL of preheated LPE while maintaining stirring at 60 °C for another 2 h. The reaction was extended further by increasing the temperature to 80 °C for 1 h. Then, the solution was allowed to cool to room temperature and centrifuged at 7000 rpm for 15 min, to obtain the settled precipitates and remove any unreacted material. Precipitates were washed three times and dried at 100 °C for 2 h. The resulting precipitates were assumed to be ZnFe_2_O_4_ NPs and stored for further characterization and application.

#### Characterization of Zinc Ferrite Nanoparticles

The confirmation of the different analytical techniques for phase, topography, and functional groups was performed for the synthesized hZnFe_2_O_4_ nano powder. The confirmation of the phase was performed using a Bruker (Karlsruhe, Germany) D-8 AXS X-ray diffractometer (XRD) operated with a rotating Cu anode with a Kα radiation source. The XRD scan speed was set to 15 rpm and was run from 10° to 80° to obtain the spectra. The crystalline size of the ZnFe_2_O_4_ NPs was calculated using the Debye–Scherrer formula. A PerkinElmer (Shelton, CT, USA) Spectrum Two Fourier-transform infrared (FTIR) spectrophotometer was used to identify the functional groups from the LPE which contributed to the synthesis of ZnFe_2_O_4_. Finally, the topographical properties of the ZnFe_2_O_4_ NPs were analyzed using a JEOL (Tokyo, Japan) JSM-IT700HR scanning electron microscope (SEM).

Dp = Kλ/βcosθ(1)

where
Dp = crystalline size of nanoparticle;
K = Scherrer constant;
λ = wavelength of incident wave;
β = full width at half maximum.

### 2.3. Synthesis of Chitosan Films

For the synthesis, 1.5 g of CH was added to three different 250 mL beakers with various ratios of LJ and LPE: 30 mL + 70 mL, 25 mL + 75 mL, and 20 mL + 80 mL. Afterwards, the mixtures were stirred at 900 rpm with simultaneous heating at 60 °C for 1 h. At the end of the process, the reaction still showed some residual CH, which did not dissolve in the beaker containing 20 mL of LJ, while in the other beakers, the CH completely dissolved. Thus, the ratio containing 25 mL of LJ was chosen for further experiments. Subsequently, 1.5 g of CH was added to a 250 mL beaker containing 25 mL of LJ and 75 mL of LPE. The mixture was stirred at 60 °C for 1 h at 900 rpm, followed by the addition of 1% glycerol as a plasticizer, to provide the films with flexibility. The glycerol was allowed to dissolve for 15 min at 400 rpm, after which the solution was removed from the hot plate [32]. No stabilizer was used, as the LJ and LPE introduced bio-compounds that caused everything to stabilize. Then, 40 mL of the prepared CH solution was poured into a 110 mm diameter Petri dish and dried overnight in a hot air oven at 45 °C. After 24 h, the CH films were carefully peeled from the Petri dishes. Figure 1 shows the schematic of the films production method.

#### Synthesis of Zinc Ferrite Nanoparticle-Containing Chitosan Films

The synthesis procedure for ZnFe_2_O_4_ NP-containing CH films was conducted with a minor modification. After 1 h of CH dissolution in the LJ and LPE solution, varying concentrations of ZnFe_2_O_4_ NPs, equivalent to 1% and 2% of the CH weight, were introduced into the solution. The mixture was stirred at 900 rpm for an additional 15 min to ensure homogeneous distribution of the NPs throughout the polymeric solution. Following the incorporation of the NPs, glycerol was added, and the mixture was stirred for another 15 min at 400 rpm. The purpose of adding glycerol was to provide the films with flexibility [32]. After the reaction was complete, 40 mL of the NPs-CH mixture was poured into a 110 mm diameter Petri dish and placed in a hot air oven for 24 h at 45 °C to form polymeric films, which were then stored for further use, as shown in Figure 1.

### 2.4. Characterization of Chitosan Films and Zinc Ferrite-Containing Chitosan Films

After obtaining the films, they were studied for various physicochemical properties. The studies included a surface morphology analysis of the films, an examination of the functional groups using FTIR, and an investigation of various mechanical and physical properties as mentioned below.

#### 2.4.1. Color Analysis

Color analysis is a major factor that defines the impact of a reinforced extract. Usually, the plant extract causes a darkening of color due to anthocyanins. For the color analysis, photos were taken of each film under homogeneous light conditions using a Canon camera. The photos of the films were cropped into 300 × 300 pixels of each photo and were analyzed for L*, a*, and b* values using ImageJ software 1.53k by NIH, USA. These values were obtained in triplicates to obtain an average of the results. From the analysis, L* indicates the lightness of the films, where a higher +ve value is lighter; a* represents a red to green shift, where a higher +ve value means a higher red shade; and b* represents a yellow to blue shift, where a higher +ve value indicates a higher yellow shade. Furthermore, ∆E defines the overall color change, and this value was calculated using the following equation [33].

(2) $$\Delta E=\left[{\left(\Delta L^{*}\right)}^{2}\right]+\left[{\left(\Delta a^{*}\right)}^{2}\right]+{\left[{\left(\Delta b^{*}\right)}^{2}\right]}^{1/2}$$

where ∆L* = L* − L*_0_,
∆a* = a* − a*_0_,
∆b* = b* − b*_0_,
where L*_0_, a*_0_, and b*_0_ are the values of a white reference, whereas L*, a*, and b* are the values of the films [33].

#### 2.4.2. Scanning Electron Microscopy and Atomic Force Microscopy

SEM and atomic force microscopy (AFM) analyses were conducted to assess the surface morphology of the generated films. Given that the films were developed using entirely natural resources, it was crucial to analyze the surface for any potential defects. Additionally, SEM was particularly important for determining whether the introduction of ZnFe_2_O_4_ induced any surface defects in the reinforced films. The analysis was performed using a JEOL JSM-IT700HR SEM. The accelerating voltage value for the electron gun was set to 3.0 kV and the photos were taken at the working distance WD = ~42 mm. The detector used was a secondary electron detector (SED). The AFM study was conducted using Park system’s AFM model XE7. A film section of 20 × 20 µm was analyzed under AFM, with a distance from the cantilever and sample set at 5 mm.

#### 2.4.3. Film Thickness

The thickness of the films significantly influences their mechanical and optical properties, with thicker films exhibiting lower transparency than thinner ones. Various factors may influence thickness, including the concentration of reinforcements and extract concentration. To assess the film thickness, measurements were taken randomly from 3 different spots using a vernier caliper. The studies were performed in triplicate to obtain a better understanding of the results. The recorded values were then averaged to confirm the overall thickness of the film.

#### 2.4.4. Fourier-Transform Infrared Spectrometry

The primary purpose of the FTIR analysis (using a PerkinElmer Spectrum Two) was to evaluate the functional groups present in the CH films and ZnFe_2_O_4_ NP-containing films. These functional groups can be from the LJ and LPE, used for the synthesis of NP-containing films and CH films. The analysis aimed to determine whether the incorporation of NPs caused any changes in the characteristic peaks of CH films compared to the bare CH film. For the experiment, small pieces of both CH film and ZnFe_2_O_4_ NP-containing film were individually placed in the FTIR spectrometer, and spectra were obtained in the range of 4000–500 cm^−1^.

#### 2.4.5. Moisture Content, Water Solubility, and Degree of Swelling

Moisture content (MC), water solubility (WS), and degree of swelling (DS) studies help in evaluating mechanical strength, degradability, and water absorption properties. If the sample has a higher WS value, it will be more degradable, whereas a higher MC value will be for a mechanically strong polymeric film. Pieces of each film sample were cut into 3 × 3 cm pieces. The weight of each piece was measured and named as M_1_. Each piece of the individual sample was placed in a 110 mm diameter Petri dish. The Petri dishes were placed inside a hot air oven for 24 h with the temperature set to 70 °C. After 24 h, the samples were removed and weighed to obtain M_2_ to measure the %MC of the samples. Later, the films were returned to the Petri dishes and each dish was filled with 25 mL distilled water. The Petri dishes were placed inside the hot air oven, but the temperature was not set, and the films were left for 24 h to obtain their M_3_ values to measure %DS. The M_3_ of the films was measured by removing them from the water. Finally, after obtaining M_3_, the films were replaced inside the hot air oven in their respective beakers and the temperature was again set to 70 °C to obtain the M_4_ values to measure their WS. All studies were triplicated to obtain an average value. The following equations were used to perform the calculations [33]:

(3) $$\mathrm{Moisture}\mathrm{content}\;(\%)=\frac{M_{1}-M_{2}}{M_{1}}\times 100$$

(4) $$\mathrm{Water}\;\mathrm{solubility}=\frac{M_{2}-M_{4}}{M_{2}}\times 100$$

(5) $$\mathrm{Degree}\mathrm{of}\mathrm{swelling}\;(\%)=\frac{M_{3}-M_{2}}{M_{2}}\times 100$$

#### 2.4.6. Water Vapor Permeability

The water vapor permeability (WVP) helps in defining the application of the developed film; for instance, WVP helps in defining how superior the barrier properties of the film will be. A lower WVP value will determine a superior barrier capability of the film. For determination of the WVP, films were sealed over 10 mL distilled water-containing cups with the help of screws and round rings, where the film was completely exposed to the outer environment. The test cups were then placed inside a desiccator and maintained an RH = 75% [34] using an environmental control chamber—forma environmental chamber 3911 (Thermo Scientific, Waltham, MA, USA). The initial weight W*_i_* of each cup was measured, before putting the cups inside the desiccator. After every 24 h for the next 72 h, the cups were removed and their final weight W*_f_* was measured. The amount of water that permeated through the films was measured using the following equation [34]:

(6) $$WVP=\left(\frac{\Delta w\;\cdot \;l}{A\;\cdot \;\Delta P\;\cdot \;t}\right)$$

where
∆*w* = weight difference (W*_i_* − W*_f_*) (g);
*l* = film thickness (m);
*A* = film area (m^2^);
∆*P* = vapor pressure difference (at 25 °C = 3170 Pa);
*t* = permeation time (s).

#### 2.4.7. Mechanical Properties

The texture analysis gives information about the mechanical strength of a sample, which helps in reaching conclusions about the application of the film samples. A sample with higher tensile strength will be better used for industrial applications for storing heavy instruments, whereas a sample with lower tensile strength will be better used for storing lighter samples. A Brookfield (Middleboro, MA, USA) CT3-10000 texture analyzer was employed to evaluate the impact of ZnFe_2_O_4_ NP incorporation into the CH films. The experimental setup involved cutting strips with dimensions of 10 mm × 60 mm from each polymeric film. The initial load was set at 0.2 N, and the sample was secured between T-96 double clamps. The final separation distance between the clamps was set to 30 mm, with a separation speed of 0.2 mm/s. The following formulas were used to calculate the stress (σ), strain (ε), and % elongation at break of the films [33]:

(7) $$\varepsilon =\left(\frac{L}{L_{0}}\right)$$

(8) $$\%E=\left(\frac{\Delta L}{L_{0}}\right)\times 100$$

(9) $$\sigma \;(\mathrm{MPa})=\frac{F}{A_{0}L_{0}}$$

#### 2.4.8. Puncture Test

The same Brookfield texture analyzer was used to test the surface strength of the film via a puncture test. In this test, stress is applied at a single point using a 0.5 mm needle, in contrast to the uniformly distributed stress across the film’s cross-section during the mechanical properties analysis. The initial load was set at 0.05 N, and the needle speed was set at 0.5 mm/s. The film sample was clamped inside a die, exposing a 5 mm area of the sample for testing. Details of the materials and methods are provided in the relevant section in the Supporting Information.

## 3. Results

### 3.1. Characterization of ZnFe_2_O_4_ Nanoparticles

**Figure 2:**
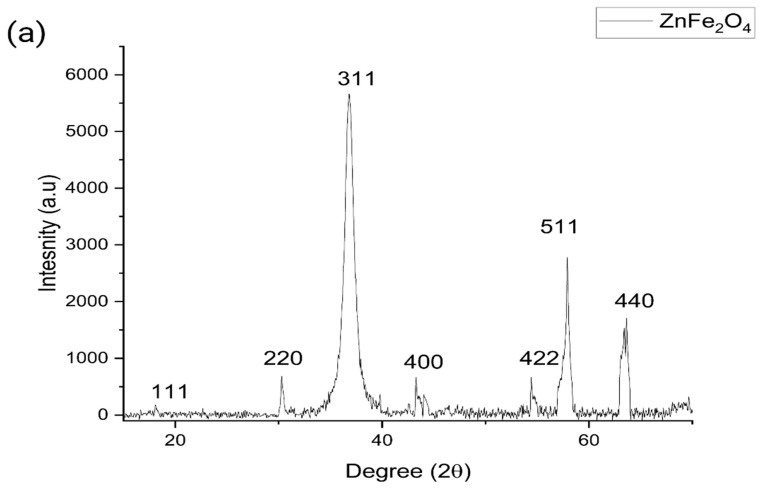

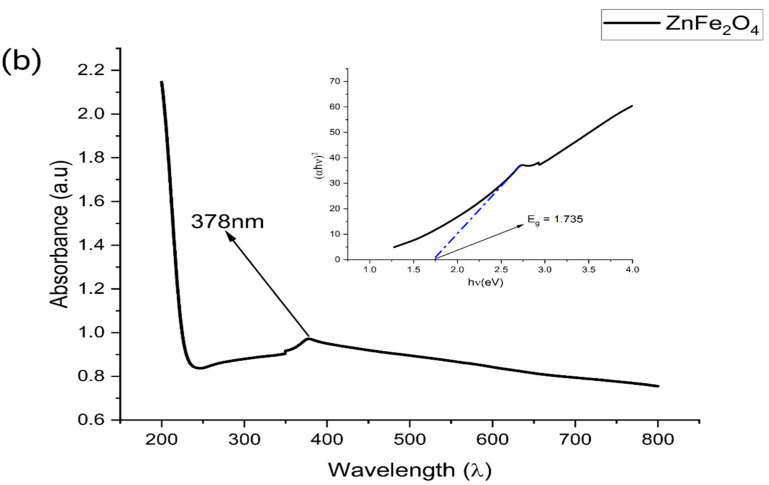
(**a**). XRD spectrum; (**b**). UV–vis spectra and inset band gap energy graph of biosynthesized ZnFe_2_O_4_ NPs.

**Figure 3:**
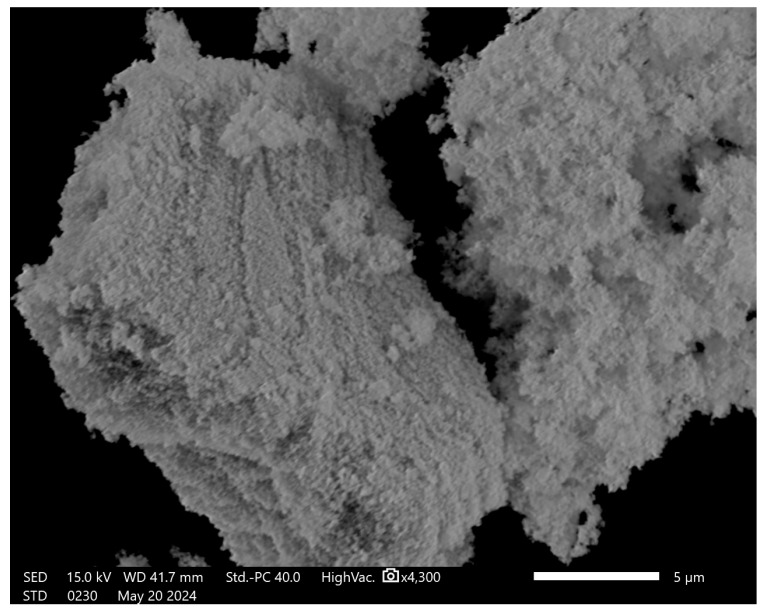
Scanning electron microscope (SEM)-captured photo of biosynthesized ZnFe_2_O_4_ NPs with sample to lens distance of ~42 mm.

**Figure 4:**
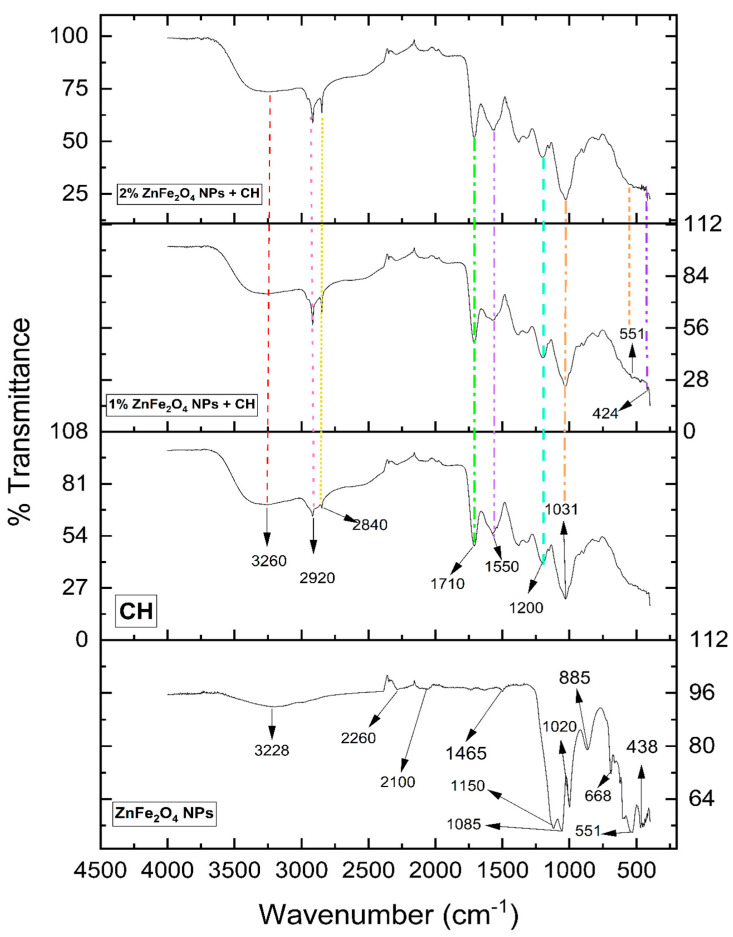
Plotted FTIR spectra for ZnFe_2_O_4_ NPs, CH, and 1% and 2% ZnFe_2_O_4_ NP-containing CH films.

The biosynthesized ZnFe_2_O_4_ NPs were found to have a cubic spinal structure [35] and all the peaks obtained in the plotted XRD spectra corresponded with Joint Committee on Powder Diffraction Standards (JCPDS) card # 022-1012 [36], as shown in Figure 2a, whereas the crystalline size calculated using the Scherrer formula was ~16 nm, as given in Table 1. The UV–visible study revealed an absorbance peak at 378 nm, and the band gap energy (E_g_) for the biosynthesized ZnFe_2_O_4_ NPs was 1.735 eV, as shown in Figure 2b. Furthermore, the SEM image revealed the rough surface of the accumulated NPs due to their magnetic nature, since it showed that the ZnF_2_O_4_ NPs were agglomerated. Furthermore, the structure is of a foamy structure type, which means that the NPs are not tightly bonded. It is known that ZnFe_2_O_4_ NPs have a superparamagnetic nature [37]. The SEM image captured for ZnFe_2_O_4_ NPs is shown in Figure 3. The FTIR results revealed peaks at 438, 551, 668, 885, 1020, 1085, 1150, 1465, 2100, 2260, and 3228 cm^−1^. The FTIR peak 438 also corresponds to the Zn–O vibration [38], whereas the peak at 511 corresponds to the Fe–O vibration [39]. Furthermore, a study suggests that the Fe–O vibration is usually found near 500 cm^−1^ [19], whereas the metal–O vibrations lie between 700 and 400 cm^−1^ [40]. Other peaks are represented in Table 2, and they could be due to the LPE, while Figure 4 shows plotted graphs of the FTIR analysis.

### 3.2. Film Thickness, Surface Morphology, and FTIR Analysis of Films

**Figure 5:**
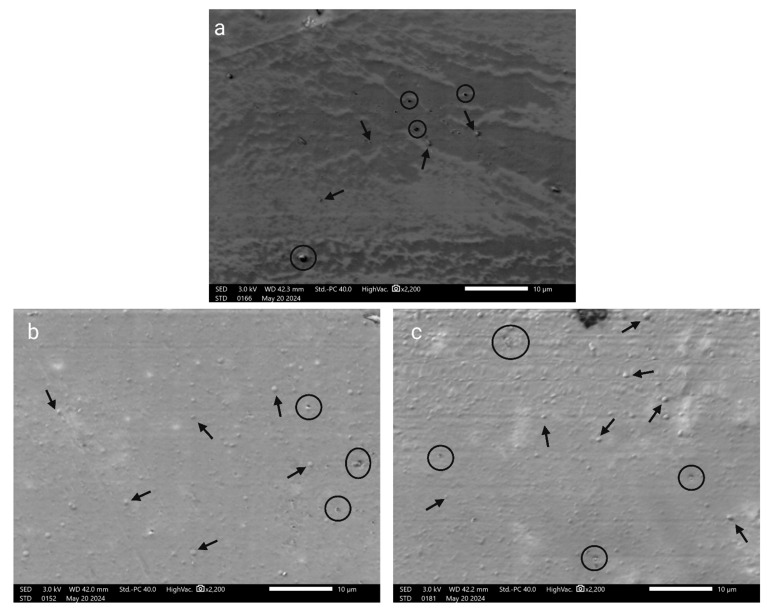
SEM-captured images of (**a**). bare CH; (**b**). 1% ZnFe_2_O_4_ NP-reinforced CH film; and (**c**). 2% ZnFe_2_O_4_ NP-reinforced CH film.

**Figure 6:**
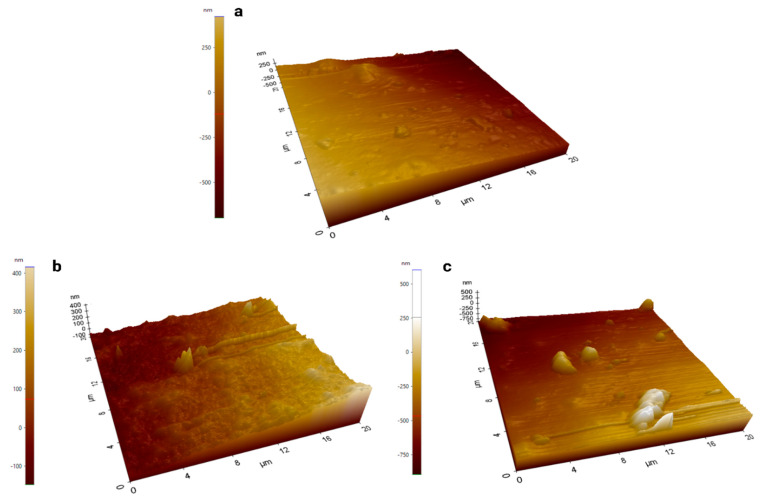
AFM images of the (**a**). bare CH film; (**b**). 1% ZnFe_2_O_4_ NP-reinforced CH film; and (**c**). 2% ZnFe_2_O_4_ NP-reinforced CH film.

The thickness of the films was almost identical, with negligible change. The thickness of the bare CH films was 0.251 ± 0.003 mm; for 1% NP-containing films, the thickness was 0.249 ± 0.002 mm; and for 2% NP-containing films, the thickness was 0.0251 ± 0.002 mm. The SEM analysis of the films, as depicted in Figure 5, includes (a) CH film, (b) CH film containing 1% ZnFe_2_O_4_ NPs, and (c) CH film containing 2% ZnFe_2_O_4_ NPs. The CH film features small, round spots across a predominantly smooth surface; these bumps likely result from the molding process where heat was applied during drying. The visible dark spots are pores, contributing to the material’s permeability to water and other substances. In Figure 5b, the CH film with 1% ZnFe_2_O_4_ NPs exhibits a rougher texture with an increased prevalence of small bumps, attributable to NPs embedded just beneath the surface, alongside smaller pores than those observed in the pure CH film. Figure 5c shows the CH film with 2% ZnFe_2_O_4_ NPs, where the surface appears rougher with a higher density of bumps, correlating with the greater NP concentration, and fewer pores overall. The pores and bumps are highlighted with circles and arrows, respectively. Further microscopic insights are provided in Figure 6, which presents AFM images of the films. Figure 6a, which displays the bare CH film, confirms its smooth surface, corroborating the SEM findings. Figure 6b reveals the surface of the CH film with 1% ZnFe_2_O_4_ NPs, characterized by a slightly rougher texture with elevated points due to the NPs. Similarly, Figure 6c, which illustrates the CH film with 2% ZnFe_2_O_4_ NPs, shows an increased number of bumps, further accentuating the impact of higher NP concentrations on surface roughness. The films showed various peaks in the FTIR spectrum. The CH film showed peaks at 1031, 1200, 1550, 1710, 2840, 2920, and 3260 cm^−1^. The correspondence of peaks is mentioned in Table 2. These peaks were common in the CH film, whereas the 1% and 2% ZnFe_2_O_4_ NP-containing CH films had two additional peaks at 424 and 551 cm^−1^, which correspond to the presence of Zn–O [41] and Fe–O [39] vibrations.

### 3.3. Study of Films’ Physical Parameters

The color analysis of the films indicated a brownish-yellow hue, attributable to the presence of anthocyanins in the LJ and LPE. Additionally, the introduction of NPs progressively darkened the color, with the shade intensifying as the concentration of NPs increased. The a* values, which indicate the shift from blue to red, were 2.746 ± 0.363 for bare CH films, 4.518 ± 0.537 for films with 1% ZnFe_2_O_4_ NPs, and 7.583 ± 0.218 for films with 2% ZnFe_2_O_4_ NPs, confirming a shift towards red with increasing NP concentration. Details of the L*, a*, b*, and ∆E values for the studied samples are provided in Table 3.

Regarding WVP, films with higher NP concentrations demonstrated enhanced barrier properties. After 72 h, the WVP values were recorded at 8.752 ± 0.015 for bare CH films, 7.578 ± 0.012 for 1% NP-containing films, and 6.299 ± 0.009 for 2% NP-containing films. This trend indicates improved barrier capabilities with increasing NP content; the values obtained are given in Table 3. Moreover, the results for %MC, %WS, and %DS showed that the films containing NPs outperformed the bare CH films in all parameters. For instance, the %WS values recorded were 37.662 ± 1.098, 54.639 ± 2.498, and 61.352 ± 1.979, respectively, for bare CH, 1%, and 2% ZnFe_2_O_4_ NP-containing films. The %WS, %DS, and %MC values improved directly in proportion to the NP concentration, underscoring the functional enhancements imparted by the NPs within the film matrix. The %MC, %DS, and %WS values for the tested films are given in Table 4. The %MC values also followed the same trend, as the NPs containing films have improved %MC; for instance, for the CH, the %MC value was 7.22 ± 1.098, and this value for 2% NP-containing CH films was 10.619 ± 0.388, which could be attributed to the hydrophilic nature of NPs [42].

### 3.4. Mechanical Properties of Films

**Figure 7:**
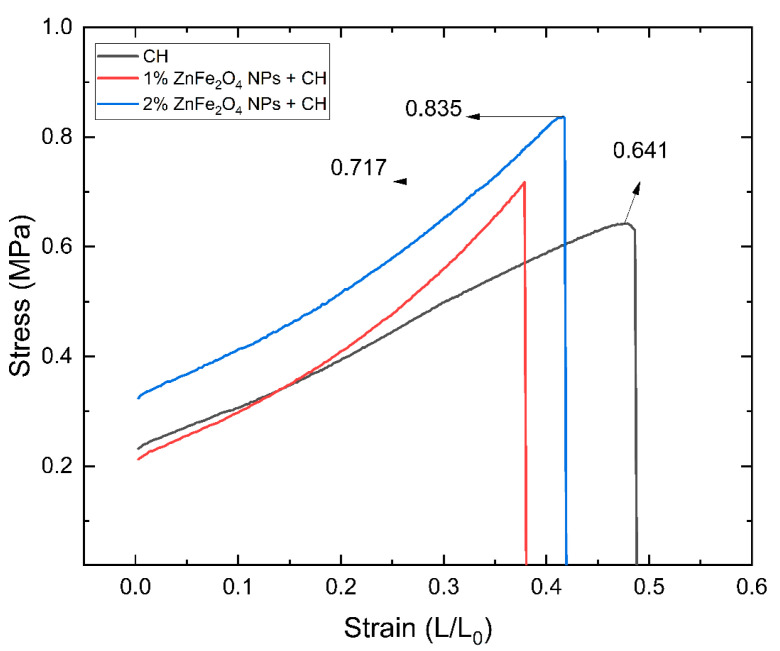
Stress–strain curves obtained for a. bare CH film; b. 1% ZnFe_2_O_4_ NP-reinforced CH film; and c. 2% ZnFe_2_O_4_ NP-reinforced CH film.

**Figure 8:**
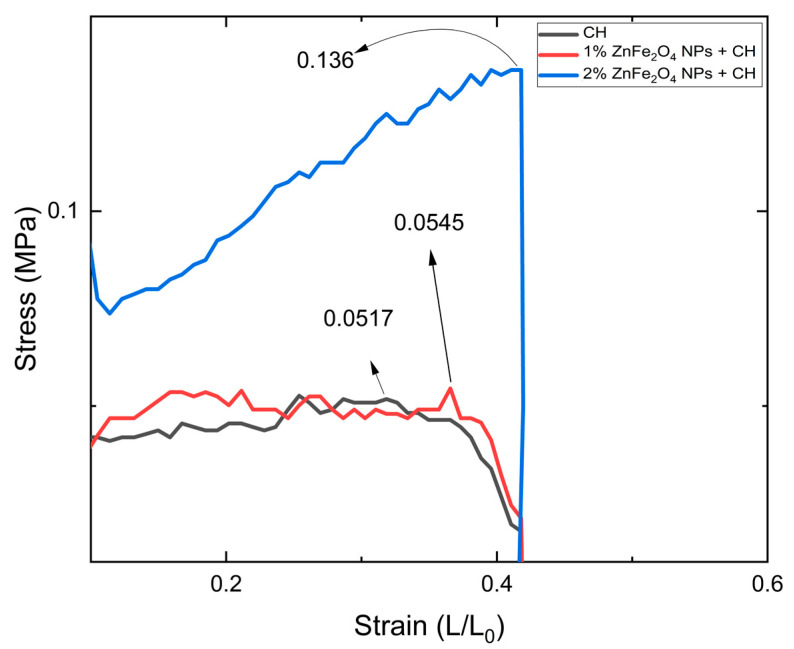
Puncture strength test plot for 1% and 2% ZnFe_2_Os CH films.

The mechanical properties of the CH films were significantly enhanced by the incorporation of ZnFe_2_O_4_ NPs. Tensile strength (TS) measurements indicated a progressive increase with higher NP concentrations: the TS for bare CH films was 0.641 MPa; for films with 1% ZnFe_2_O_4_ NPs, it was 0.717 MPa; and for films with 2% ZnFe_2_O_4_ NPs, it reached 0.835 MPa, as shown in Figure 7. Conversely, the elongation at break (%E) values demonstrated a decrease with the addition of NPs: 39.843% for bare CH, 32.678% for 1% ZnFe_2_O_4_ NPs, and 29.853% for 2% ZnFe_2_O_4_ NPs. The Young’s modulus (YM) also increased, indicating a stiffer film structure with the addition of NPs. The YM values were 1.346 MPa for bare CH films, 1.896 MPa for 1% NP-containing films, and 2.007 MPa for 2% NP-containing films. Furthermore, the puncture strength of the films improved, reflecting a stronger surface due to NP inclusion, with values of 0.051 MPa for bare CH, 0.054 MPa for 1% ZnFe_2_O_4_ NPs, and a significant increase to 0.136 MPa for 2% ZnFe_2_O_4_ NPs; the plotted results are shown in Figure 8. These results highlight the reinforcement effect of ZnFe_2_O_4_ NPs on the mechanical integrity of CH films. Table 4 contains the data values recorded for TS, YM, %E, and puncture strength for all the tested samples.

## 4. Discussion

The ZnFe_2_O_4_ NPs were biosynthesized using LPE, and the phase of NPs was confirmed via XRD analysis, aligning with JCPDS card # 022–1012 [36], with a cubic spinal crystal structure. By employing FWHM, the crystalline size of the ZnFe_2_O_4_ NPs was computed to be ~16 nm. Furthermore, the small and sharp peaks indicate that the biosynthesized NPs have better crystallinity [43]. Furthermore, the SEM analysis revealed rough-surfaced agglomerated NPs, for which there are two possible reasons: first, the magnetic nature of the ZnFe_2_O_4_ NPs, as they are known to have a superparamagnetic nature [44]; and secondly, the annealing temperature, as it is known that the annealing of NPs causes an increase in crystalline size [45]. Furthermore, a furry surface can be detected from the SEM image, which could be due to weaker bonding, which could be beneficial when introducing the NPs inside the CH polymer matrix, as it would be easier for them to separate. Further, the FTIR analysis revealed vibrational peaks 438 and 551 cm^−1^, which, according to the literature correspond to Zn–O [41] and Fe–O [39] stretching, respectively, underscoring the successful incorporation of metal ions into the NP structure.

It is also worth noting that the introduction of NPs did not affect the film thickness, as the amount of the ZnFe_2_O_4_ NPs used was quite small, i.e., 1 and 2 wt.% of CH. The variation in thickness could play a part in manipulating the mechanical properties of the films [46], which could make it difficult to study the impact of NPs to analyze the mechanical properties. Furthermore, the thicker the film, the less transparent it becomes, as the film has a red hue. This stability is crucial for maintaining consistent performance in applications such as packaging or barrier films where dimensional stability can influence mechanical and barrier properties [47].

The SEM images show that the surface of the films became rougher with the increase in NP reinforcement. The apparent roughness could be attributed to two phenomena, namely, molding, and NPs being trapped under the surface. Since the drying conditions were the same, molding methods cannot have much impact on the surface as only a few bumps are seen on the bare CH films, while the NP concentration could justify the increase in the number of surface bumps. The analysis shows that the majority of NPs are under the film surface, since they are not visible on the surface. AFM analyses of CH films with embedded ZnFe_2_O_4_ NPs also demonstrated an increase in surface roughness proportional to NP content. Furthermore, the NPs can act as nanofillers, which can create the appearance of bumps and increased peaks [48], whereas the magnetic nature of the NPs can also cause NP accumulation, potentially leading to the presence of higher and broader peaks on the NP-containing CH films. This could increase the mechanical interlocking in composite materials, enhancing the mechanical properties, as evidenced by the increase in tensile strength and YM with higher NP concentrations. Conversely, the decrease in %elongation at the break point with increasing NP content highlights a trade-off between strength and flexibility, a common theme in composite material design.

Moreover, the color analysis results reflect the influence of NP content on the optical properties of the films. The presence of anthocyanins from LJ and LPE produced a brownish-red color with a yellow shade. It is well known that the heat causes a brown to red shift in the anthocyanin color [49]. Furthermore, the ZnFe_2_O_4_ NPs have a brick-red color as well, and their presence could cause the darkening of the color, as evident from the color analysis, which showed that increasing NP concentration shows a shift towards red, as in the a* values. Further, shifting towards a darker, redder hue with increasing ZnFe_2_O_4_ NP concentration could be advantageous in applications requiring UV protection or specific aesthetic qualities, as evident from the L* values, which became darker. The L* value for CH films was recorded as 49.601 ± 2.350; for 1% ZnFe_2_O_4_ NPs, it was 43.925 ± 1.347; and for 2% ZnFe_2_O_4_ NPs, it was 41.792 ± 1.319, as, if the L* value is closer to 100, the film becomes lighter [50].

The ZnFe_2_O_4_ NPs are thought to occupy the vacant spaces in the CH polymeric structure, thus strengthening it and improving the barrier properties [51]. The study of WVP with increased NP content illustrates the potential of ZnFe_2_O_4_ NPs to enhance barrier properties in films. The obtained results show that when a higher concentration of NPs is enhanced, they tend to block the pores, causing a decline in water permeation, as the WVP value for the CH films after 72 h was 8.752 ± 0.015 × 10^−10^ g.mm/m^2^.d.kPa; for 1% ZnFe_2_O_4_ NP-containing CH films, it was 7.578 ± 0.012 × 10^−10^ g.mm/m^2^.d.kPa; and for 2% ZnFe_2_O_4_ NPs, it was 6.299 ± 0.009 × 10^−10^ g.mm/m^2^.d.kPa. This could be particularly beneficial in food packaging applications where moisture barrier enhancement is needed to improve shelf life. The observed changes in MC, WS, and DS further corroborate the functional enhancement of the CH films, aligning with the goal of achieving tailored material properties through NP incorporation. However, these films are not recommended for their application in aquatic environments, since the CH is water-soluble, and the film structure can be destroyed easily.

Furthermore, the hypothesis that NPs were occupying vacant spaces in polymeric structures and providing structural strength was proven by the mechanical properties study [52]. The results showed an increase in tensile strength from 0.641 to 0.835 MPa after the introduction of 2% ZnFe_2_O_4_ NPs to the CH polymeric structure. Although the mechanical strength was increasing by occupying the vacancies, it was also making the structure rigid, causing a decrease in %E values from 39.843 to 29.853 on the introduction of 2% ZnFe_2_O_4_ NPs, limiting their application where flexibility is needed. Furthermore, the homogeneously distributed NPs cause the bonding with the CH polymeric chains and result in bringing the polymeric chains together and closer, hence distributing the applied load, improving the mechanical strength [53]. The puncture strength revealed the same properties; as with the introduction of ZnFe_2_O_4_ NPs, it caused an increase in the surface strength.

In summary, the integration of ZnFe_2_O_4_ NPs into CH films not only preserves but also enhances the mechanical and barrier properties of the films, confirming the potential of this nanocomposite approach in advanced material applications. Future studies could explore the long-term stability of these properties and the potential environmental impacts of NP leaching or degradation, ensuring the sustainable application of these advanced materials. Table 5 presents a brief literature review to compare the results obtained in this study with those previously reported. Overall, the current work shows better mechanical strength compared to the 0.04 MPa value reported by Jancikova, S et al. [54]

## 5. Conclusions

This study effectively developed sustainable CH films using natural acids (LJ and LPE), introducing a green synthesis approach for enhanced mechanical and barrier properties through ZnFe_2_O_4_ NP reinforcement. The incorporation of ZnFe_2_O_4_ NPs increased film thickness marginally but enhanced tensile strength and puncture resistance significantly, making the films stiffer and more robust, though slightly lowering their flexibility. The surface roughness also increased, primarily due to embedded NPs, improving interlocking between the matrix and nanofillers. The films exhibited a reddish hue, which intensified with higher NP concentrations, indicating likely suitability for UV shielding or aesthetic applications. WVP decreased with NP content, highlighting improved barrier properties appropriate for food packaging, while higher WS and MC point to biodegradability, which is desirable for environmentally friendly applications. Overall, ZnFe_2_O_4_ NP reinforcement in CH films aligns with sustainability goals, offering favorable applications in packaging and medical products, meeting the demand for eco-friendly and high-performance materials.

## Figures and Tables

**Table 1:** Crystaline size calculation of biosynthesized ZnFe_2_O_4_ NPs using Scherrer equation.

hkl	Peak Position (2θ)	FWHM	Size (nm)
111	18.041	0.479	17.53
220	30.331	0.282	30.46
311	36.832	1.218	7.17
400	43.634	1.084	8.24
422	54.574	0.482	19.36
511	57.871	0.701	13.52
400	63.459	0.727	13.41
**Average Crystalline Size**			**15.67**

**Table 2:** FTIR spectrum peaks and their correspondence.

FTIR Peak Position	Correspondence	FTIR Peak Position	Correspondence
424—NP Films	Zn–O vibration	1465—ZnFe_2_O_4_ NPs	C–H bending
438—ZnFe_2_O_4_ NPs	Zn–O vibration	1550—CH + NP Films	N–O stretching
551—NP Films + NPs	Fe–O vibration	1710—CH + NP Films	C=O stretching
668—ZnFe_2_O_4_ NPs	C=C bending	2100—ZnFe_2_O_4_ NPs	C≡C stretching
885—ZnFe_2_O_4_ NPs	C=C bending	2260—ZnFe_2_O_4_ NPs	C≡C stretching
1020—ZnFe_2_O_4_ NPs	C–O stretching	2840—CH + NP Films	C–H stretching
1031—CH + NP Films	C–O stretching	2920—CH + NP Films	O–H stretching
1085—ZnFe_2_O_4_ NPs	C–O stretching	3228—CH + NP Films	O–H stretching
1150—ZnFe_2_O_4_ NPs	C–O stretching	3260—ZnFe_2_O_4_ NPs	O–H stretching
1200—CH + NPs Films	C–O stretching		

**Table 3:** L, a, b, ∆E, and WVP values recorded for bare CH, 1%, and 2% ZnFe_2_O_4_ NP-containing CH films.

Sample	L*	a*	b*	∆E	WVP (×10^−10^ g.mm/m^2^·d·kPa)		
					24 h	48 h	72 h
**CH**	49.601 ± 2.350	2.746 ± 0.363	34.769 ± 1.576	34.995 ± 0.973	2.568 ± 0.007	5.505 ± 0.011	8.752 ± 0.015
**1% ZnFe_2_O_4_ NPs CH**	43.925 ± 1.347	4.518 ± 0.537	36.472 ± 2.058	40.957 ± 1.839	2.062 ± 0.009	4.607 ± 0.008	7.578 ± 0.012
**2 ZnFe_2_O_4_ NPs CH**	41.792 ± 1.319	7.583 ± 0.218	32.508 ± 1.681	41.169 ± 1.666	1.885 ± 0.010	4.233 ± 0.009	6.299 ± 0.009

**Table 4:** The %MC, %WS, %DS, TS, YM, %E, and puncture strength values for bare CH films, 1%, and 2% ZnFe_2_O_4_ NP-containing CH films.

Sample	%MC	%WS	%DS	YM (MPa)	%E	TS (MPa)	Puncture Test (MPa)
**CH**	7.228 ± 0.308	37.662 ± 1.098	9.740 ± 0.490	1.346	39.843	0.641	0.051
**1% ZnFe_2_O_4_ NPs CH**	8.334 ± 0.293	54.639 ± 2.498	13.526 ± 0.513	1.896	32.678	0.717	0.053
**2 ZnFe_2_O_4_ NPs CH**	10.619 ± 0.388	61.352 ± 1.979	17.247 ± 0.839	2.007	29.853	0.835	0.136

**Table 5:** Literature review values compared with current study values.

Polymer	Plant Extract	Nanoparticles	L*	a*	b*	TS (MPa)	Reference
CH + PVA	-	Fe_2_O_3_	-	-	-	8.46	[55]
CH + Pectin	-	Fe_3_O_4_	-	-	-	40.1	[56]
CH	*Brassica oleracea var.* *capitata f. Rubra*	-				0.04	[54]
=	*Ipomoea batatas*					2.32	[54]
=	*Clitoria* *ternatea*					3.12	[54]
CH	Soy lecithin + tea tree essential oil		90.16	−0.80	7.30	4.09	[57]
CH + PVA	Lemon extract (0.01%)	-	73.17	3.95	31.48	6.08	[58]
CH	Mango leaf extract (1%)	-	86.38	1.08	1.83	~19	[34]
CH	LPE (75%)	ZnFe_2_O_4_	34.792	7.583	32.508	0.835	Current work

## Data Availability

The original contributions presented in the study are included in the article/Supplementary Material, further inquiries can be directed to the corresponding author.

## Supplementary Materials

The following supporting information can be downloaded at: https://www.mdpi.com/article/10.3390/polym16243466/s1, Figure S1. a. XRD spectrum, b. UV vis spectra and inset band gap energy graph of biosynthesized ZnFe_2_O_4_ NPs. Figure S2. SEM captured photo of biosynthesized ZnFe_2_O_4_ NPs with sample to lens distance of ~42 mm. Figure S3. Plotted FTIR spectra for ZnFe_2_O_4_ NPs, Ch, and 1% and 2% ZnFe_2_O_4_ NPs containing CH films. Figure S4. Puncture strength test plot for CH, 1% and 2% ZnFe_2_O_s_ CH films. Table S1. Crystaline size calculation of biosynthesized ZnFe_2_O_4_ NPs using Scherrer equation. Table S2. FTIR spectrum peaks and their correspondence. Table S3. %MC, %WS, %DS, TS, YM, %E and puncture strength values for bare CH films, 1% and 2% ZnFe_2_O_4_ NPs containing CH films.

## References

[1] Das O., Babu K., Shanmugam V., Sykam K., Tebyetekerwa M., Neisiany R.E., Försth M., Sas G., Gonzalez-Libreros J., Capezza A.J. (2022). Natural and industrial wastes for sustainable and renewable polymer composites. Renew. Sustain. Energy Rev..

[2] Geyer R., Jambeck J.R., Law K.L. (2017). Production, use, and fate of all plastics ever made. Sci. Adv..

[3] Liu X., Liao W., Xia W. (2023). Recent advances in chitosan based bioactive materials for food preservation. Food Hydrocoll..

[4] Li X., Lin Y., Liu M., Meng L., Li C. (2023). A review of research and application of polylactic acid composites. J. Appl. Polym. Sci..

[5] Verma P., Rani R., Das D., Rai K.K., Gogoi P., Badwaik L.S. (2024). Transformation of banana peel into biodegradable film added with starch and carboxymethyl cellulose and its characterization. Sustain. Chem. Pharm..

[6] Vidal J.L., Yavitt B.M., Wheeler M.D., Kolwich J.L., Donovan L.N., Sit C.S., Hatzikiriakos S.G., Jalsa N.K., MacQuarrie S.L., Kerton F.M. (2022). Biochar as a sustainable and renewable additive for the production of Poly(ε-caprolactone) composites. Sustain. Chem. Pharm..

[7] Mukherjee C., Varghese D., Krishna J.S., Boominathan T., Rakeshkumar R., Dineshkumar S., Brahmananda Rao C.V.S., Sivaramakrishna A. (2023). Recent advances in biodegradable polymers—Properties, applications and future prospects. Eur. Polym. J..

[8] Wang Z., Zhou X., Sheng L., Zhang D., Zheng X., Pan Y., Yu X., Liang X., Wang Q., Wang B. (2023). Effect of ultrasonic degradation on the structural feature, physicochemical property and bioactivity of plant and microbial polysaccharides: A review. Int. J. Biol. Macromol..

[9] Wahab D.N.A., Siddique M.B.M., Chew J.J., Su H.T., Khairuddin N., Khaerudini D.S., Hossain M.S., Sunarso J. (2023). Characterization of starch biofilm reinforced with cellulose microfibers isolated from Musa Saba’ midrib residue and its application as an active packaging film. J. Appl. Polym. Sci..

[10] Grzybek P., Dudek G., van der Bruggen B. (2024). Cellulose-based films and membranes: A comprehensive review on preparation and applications. J. Chem. Eng.

[11] Islam M.M., Shahruzzaman M., Biswas S., Nurus Sakib M., Rashid T.U. (2020). Chitosan based bioactive materials in tissue engineering applications—A review. Bioact. Mater..

[12] El Mejri W., El Mahdi A., Mendes F., Castro R., Njim L., Zaied S., Tomás H., Baati T., Medimagh R., Khwaldia K. (2024). Chitosan films synthesized via thiol-ene click chemistry: Toward safe and versatile platforms for packaging, cosmetics and biomedical applications. Sustain. Chem. Pharm..

[13] Qiao C., Ma X., Wang X., Liu L. (2021). Structure and properties of chitosan films: Effect of the type of solvent acid. LWT.

[14] Olsson E., Hedenqvist M.S., Johansson C., Järnström L. (2013). Influence of citric acid and curing on moisture sorption, diffusion and permeability of starch films. Carbohydr. Polym..

[15] Sani A., Murad A., Hassan D., Channa G.M., El-Mallul A., Medina D.I. (2023). Photo-catalytic and biomedical applications of one-step, plant extract-mediated green-synthesized cobalt oxide nanoparticles. Environ. Sci. Pollut. Res..

[16] Dhaka A., Chand Mali S., Sharma S., Trivedi R. (2023). A review on biological synthesis of silver nanoparticles and their potential applications. Results Chem..

[17] Xu L., Wang Y.-Y., Huang J., Chen C.-Y., Wang Z.-X., Xie H. (2020). Silver nanoparticles: Synthesis, medical applications and biosafety. Theranostics.

[18] Khan M.A.R., Al Mamun M.S., Habib M.A., Islam A.B.M.N., Mahiuddin M., Karim K.M.R., Naime J., Saha P., Dey S.K., Ara M.H. (2022). A review on gold nanoparticles: Biological synthesis, characterizations, and analytical applications. Results Chem..

[19] Hassan D., Khalil A.T., Saleem J., Diallo A., Khamlich S., Shinwari Z.K., Maaza M. (2018). Biosynthesis of pure hematite phase magnetic iron oxide nanoparticles using floral extracts of Callistemon viminalis (bottlebrush): Their physical properties and novel biological applications. Artif. Cells Nanomed. Biotechnol..

[20] Boura-Theodoridou O., Giannakas A., Katapodis P., Stamatis H., Ladavos A., Barkoula N.-M. (2020). Performance of ZnO/chitosan nanocomposite films for antimicrobial packaging applications as a function of NaOH treatment and glycerol/PVOH blending. Food Packag. Shelf Life.

[21] Ardebilchi Marand S., Almasi H., Ardebilchi Marand N. (2021). Chitosan-based nanocomposite films incorporated with NiO nanoparticles: Physicochemical, photocatalytic and antimicrobial properties. Int. J. Biol. Macromol..

[22] Dong W., Su J., Chen Y., Xu D., Cheng L., Mao L., Gao Y., Yuan F. (2022). Characterization and antioxidant properties of chitosan film incorporated with modified silica nanoparticles as an active food packaging. Food Chem..

[23] Kloster G.A., Mosiewicki M.A., Marcovich N.E. (2019). Chitosan/iron oxide nanocomposite films: Effect of the composition and preparation methods on the adsorption of congo red. Carbohydr. Polym..

[24] Xing Q., Buono P., Ruch D., Dubois P., Wu L., Wang W.-J. (2019). Biodegradable UV-Blocking Films through Core–Shell Lignin–Melanin Nanoparticles in Poly(butylene adipate-co-terephthalate). ACS Sustain. Chem. Eng..

[25] Jafarova V.N., Orudzhev G.S. (2021). Structural and electronic properties of ZnO: A first-principles density-functional theory study within LDA(GGA) and LDA(GGA)+U methods. Solid State Commun..

[26] Saleem S., Ashiq M.N., Manzoor S., Ali U., Liaqat R., Algahtani A., Mujtaba S., Tirth V., Alsuhaibani A.M., Refat M.S. (2023). Analysis and characterization of opto-electronic properties of iron oxide (Fe_2_O_3_) with transition metals (Co, Ni) for the use in the photodetector application. J. Mater. Res. Technol..

[27] Roumaih K., Ismail S.M., Labib S., Helal A. (2023). Structural, magnetic, and optical properties of ZnFe2O4/RO (RO = CdO, NiO, Ga2O3, SnO2, and TiO2) nanocomposites. J. Mater. Sci..

[28] Nyabadza A., McCarthy É., Makhesana M., Heidarinassab S., Plouze A., Vazquez M., Brabazon D. (2023). A review of physical, chemical and biological synthesis methods of bimetallic nanoparticles and applications in sensing, water treatment, biomedicine, catalysis and hydrogen storage. Adv. Colloid Interface Sci..

[29] Egbuna C., Parmar V.K., Jeevanandam J., Ezzat S.M., Patrick-Iwuanyanwu K.C., Adetunji C.O., Khan J., Onyeike E.N., Uche C.Z., Akram M. (2021). Toxicity of Nanoparticles in Biomedical Application: Nanotoxicology. J. Toxicol..

[30] Pourmadadi M., Aslani A., Hassan D., Sani A., Rahdar A.I., Medina D., Abdouss M., Romanholo Ferreira L.F. (2023). Recent advancements in the targeted delivery of Gemcitabine: Harnessing nanomedicine for enhanced cancer therapy. OpenNano.

[31] Sani A., Hassan D., Khalil A.T., Mughal A., El-Mallul A., Ayaz M., Yessimbekov Z., Shinwari Z.K., Maaza M. (2021). Floral extracts-mediated green synthesis of NiO nanoparticles and their diverse pharmacological evaluations. J. Biomol. Struct. Dyn..

[32] Leceta I., Guerrero P., de la Caba K. (2013). Functional properties of chitosan-based films. Carbohydr. Polym..

[33] Hernández-Varela J.D., Chanona-Pérez J.J., Resendis-Hernández P., Gonzalez Victoriano L., Méndez-Méndez J.V., Cárdenas-Pérez S., Calderón Benavides H.A. (2022). Development and characterization of biopolymers films mechanically reinforced with garlic skin waste for fabrication of compostable dishes. Food Hydrocoll..

[34] Rambabu K., Bharath G., Banat F., Show P.L., Cocoletzi H.H. (2019). Mango leaf extract incorporated chitosan antioxidant film for active food packaging. Int. J. Biol. Macromol..

[35] Andhare D.D., Jadhav S.A., Khedkar M.V., Somvanshi S.B., More S.D., Jadhav K.M. (2020). Structural and Chemical Properties of ZnFe2O4 Nanoparticles Synthesised by Chemical Co-Precipitation Technique. J. Phys. Conf. Ser..

[36] Yadav N.G., Chaudhary L.S., Sakhare P.A., Dongale T.D., Patil P.S., Sheikh A.D. (2018). Impact of collected sunlight on ZnFe_2_O_4_ nanoparticles for photocatalytic application. J. Colloid Interface Sci..

[37] Li F., Wang H., Wang L., Wang J. (2007). Magnetic properties of ZnFe_2_O_4_ nanoparticles produced by a low-temperature solid-state reaction method. J. Magn. Magn. Mater..

[38] Karthik K.V., Raghu A.V., Reddy K.R., Ravishankar R., Sangeeta M., Shetti N.P., Reddy C.V. (2022). Green synthesis of Cu-doped ZnO nanoparticles and its application for the photocatalytic degradation of hazardous organic pollutants. Chemosphere.

[39] Abdullah J.A.A., Salah Eddine L., Abderrhmane B., Alonso-González M., Guerrero A., Romero A. (2020). Green synthesis and characterization of iron oxide nanoparticles by pheonix dactylifera leaf extract and evaluation of their antioxidant activity. Sustain. Chem. Pharm..

[40] Mattox D.M., Mattox D.M. (2010). Chapter 2—Substrate (“Real”) Surfaces and Surface Modification. Handbook of Physical Vapor Deposition (PVD) Processing.

[41] Joesna G., Saravanan P., Ferin R.Z., Gunachitra T., Sankar D., Tamilselvan S., Meena M., SenthilKannan K., Vimalan M., Mohamed M.G. (2022). Domestic microwave supported green synthesis of ZnO nanoparticles for electronic, mechano, rheological and frequency intensifying applications. J. Mater. Sci. Mater. Electron..

[42] Chang C.-I., Lee W.-J., Young T.-F., Ju S.-P., Chang C.-W., Chen H.-L., Chang J.-G. (2008). Adsorption mechanism of water molecules surrounding Au nanoparticles of different sizes. J. Chem. Phys..

[43] Thygesen A., Oddershede J., Lilholt H., Thomsen A.B., Ståhl K. (2005). On the determination of crystallinity and cellulose content in plant fibres. Cellulose.

[44] Gazzola G., Ambrosetti M., Mozzati M.C., Albini B., Galinetto P., Bini M. (2021). Tuning the superparamagnetic effect in ZnFe2O4 nanoparticles with Mg, Ga doping. Mater. Chem. Phys..

[45] Alluqmani S.M., Loulou M., Ouerfelli J., Alshahrie A., Salah N. (2021). Annealing effect on structural and optical properties of nanostructured carbon of oil fly ash modified titania thin-film. Results Phys..

[46] Saito M., Ito K., Yokoyama H. (2022). Film thickness and strain rate dependences of the mechanical properties of polystyrene-b-polyisoprene-b-polystyrene block copolymer ultrathin films forming a spherical domain. Polymer.

[47] Lai W.-F., Wong W.-T. (2022). Design and Practical Considerations for Active Polymeric Films in Food Packaging. Int. J. Mol. Sci..

[48] Pires J., Paula C.D.d., Souza V.G.L., Fernando A.L., Coelhoso I. (2021). Understanding the Barrier and Mechanical Behavior of Different Nanofillers in Chitosan Films for Food Packaging. Polymers.

[49] Rhim J.W., Nunes R.V., Jones V.A., Swartzel K.R. (1989). Kinetics of Color Change of Grape Juice Generated using Linearly Increasing Temperature. J. Food Sci..

[50] Becker D., Becker D. (2016). 37—Color Measurement. Color Trends and Selection for Product Design.

[51] de Moura M.R., Lorevice M.V., Mattoso L.H.C., Zucolotto V. (2011). Highly Stable, Edible Cellulose Films Incorporating Chitosan Nanoparticles. J. Food Sci..

[52] Jamróz E., Kulawik P., Kopel P. (2019). The Effect of Nanofillers on the Functional Properties of Biopolymer-Based Films: A Review. Polymers.

[53] Upadhyay P., Ullah A. (2024). Enhancement of mechanical and barrier properties of chitosan-based bionanocomposites films reinforced with eggshell-derived hydroxyapatite nanoparticles. Int. J. Biol. Macromol..

[54] Jancikova S., Dordevic D., Tesikova K., Antonic B., Tremlova B. (2021). Active Edible Films Fortified with Natural Extracts: Case Study with Fresh-Cut Apple Pieces. Membranes.

[55] Hoque M.A., Ahmed M.R., Rahman G.T., Rahman M.T., Islam M.A., Khan M.A., Hossain M.K. (2018). Fabrication and comparative study of magnetic Fe and α-Fe2O3 nanoparticles dispersed hybrid polymer (PVA + Chitosan) novel nanocomposite film. Results Phys..

[56] Zarandona I., Correia D.M., Moreira J., Costa C.M., Lanceros-Mendez S., Guerrero P., de la Caba K. (2023). Magnetically responsive chitosan-pectin films incorporating Fe3O4 nanoparticles with enhanced antimicrobial activity. Int. J. Biol. Macromol..

[57] Cazón P., Antoniewska A., Rutkowska J., Vázquez M. (2021). Evaluation of easy-removing antioxidant films of chitosan with Melaleuca alternifolia essential oil. Int. J. Biol. Macromol..

[58] Wang D., Shao S., Wang B., Guo D., Tan L., Chen Q. (2024). Fabrication of chitosan/guar gum/polyvinyl alcohol films incorporated with polymethoxyflavone-rich citrus extracts: Postharvest shelf-life extension of strawberry fruits. Prog. Org. Coat..

