# Supplementary material for: Environmentally Sustainable and Green Polymeric Method for Chitosan (CH) Film Synthesis Using Natural Acids and Impact of Zinc Ferrite Nanoparticles (NPs) on Water Solubility (WS) and Physical Properties
Source: Polymers (Basel). 2024 Dec 12;16(24):3466. doi: 10.3390/polym16243466 (PMC11728712; doi:10.3390/polym16243466)
Supplement: Supplementary file 1 [file polymers-16-03466-s001.zip › polymers-3294214-supplementary.pdf]

Environmentally Sustainable and Green Polymeric Method for Chitosan (CH) Film Synthesis Using Natural Acids and Impact of Zinc Ferrite Nanoparticles (NPs) on Water Solubility (WS) and Physical Properties

Dilawar Hassan [1,*], Ayesha Sani [1], Ghulam Qadir Chanihoon [2], Aurora Antonio Pérez [1], Muhammad Ehsan [3] and Ana Laura Torres Huerta [1]

1 School of Engineering and Sciences, Tecnologico de Monterrey, Atizapan de Zaragoza, Estado de Mexico C.P. 52926, Mexico

2 National Centre of Excellence in Analytical Chemistry (NCEAC), University of Sindh, Jamshoro 76080, Pakistan

3 Centro de Bachillerato Tecnológico Agropecuario, 162. Carr. Mexico-Veracruz Vía Texcoco km 95, Francisco I. Madero, Tlaxcala C. P. 90280, Mexico

* Correspondence: a01754343@tec.mx

SUPPLEMMENTRY SECTION

Figures:

Figure S1. a. XRD spectrum, b. UV vis spectra and inset band gap energy graph of biosynthesized $ZnFe_2O_4$ NPs.

Figure S2. SEM captured photo of biosynthesized $ZnFe_2O_4$ NPs with sample to lens distance of ~42mm.

Figure S3. Plotted FTIR spectra for ZnFe2O4 NPs, Ch, and 1% and 2% ZnFe2O4 NPs containing CH films.

Figure S4. Puncture strength test plot for CH, 1% and 2% $ZnFe_2O_s$ CH films.

Tables:

Table S1. Crystaline size calculation of biosynthesized $ZnFe_2O_4$ NPs using Scherrer equation.

Table S2. FTIR spectrum peaks and their correspondence.

Table S3. %MC, %WS, %DS, TS, YM, %E and puncture strength values for bare CH films, 1% and 2% $ZnFe_2O_4$ NPs containing CH films

Figures:

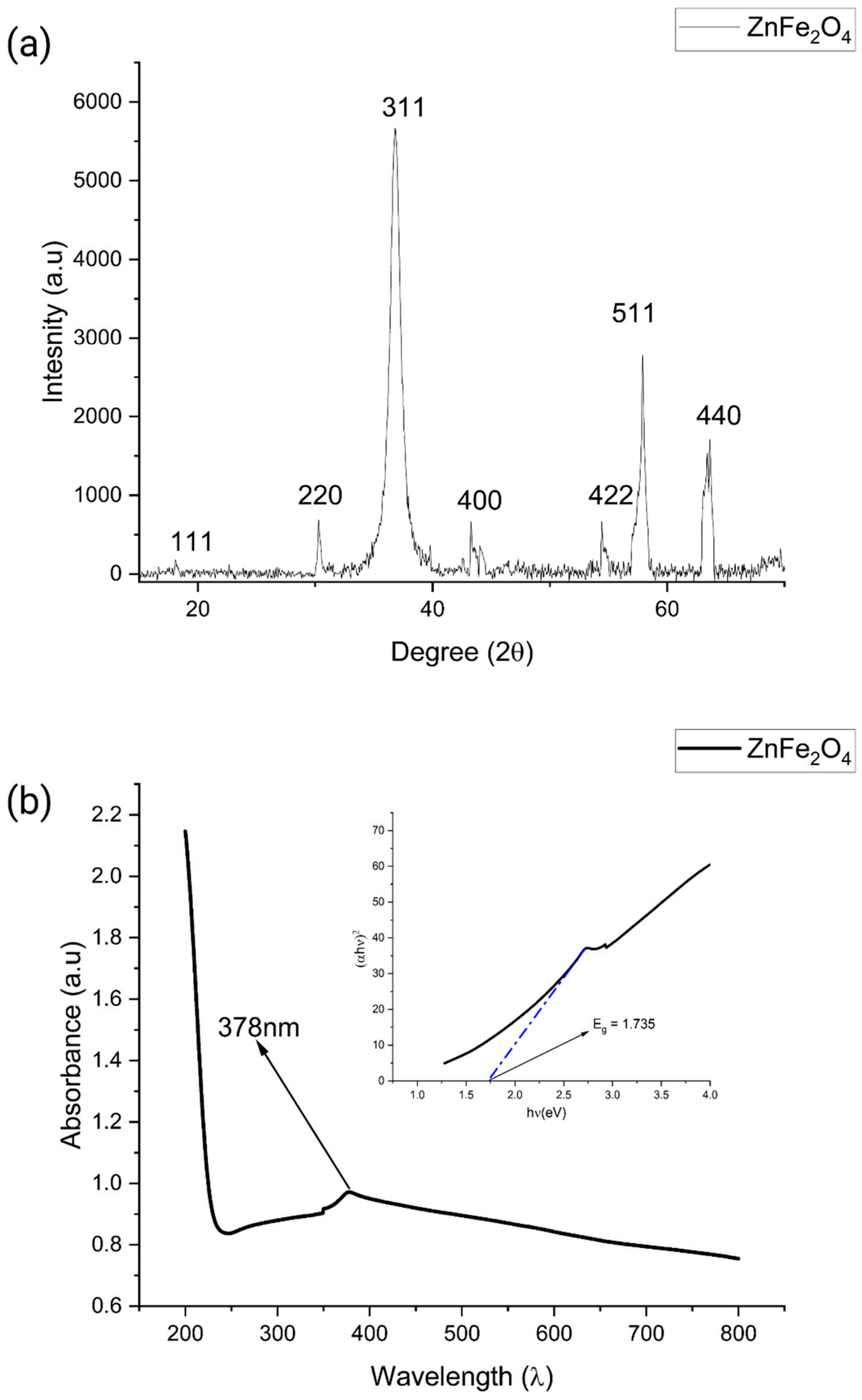

Figure S1. a. XRD spectrum, b. UV vis spectra and inset band gap energy graph of biosynthesized $ZnFe_2O_4$ NPs.

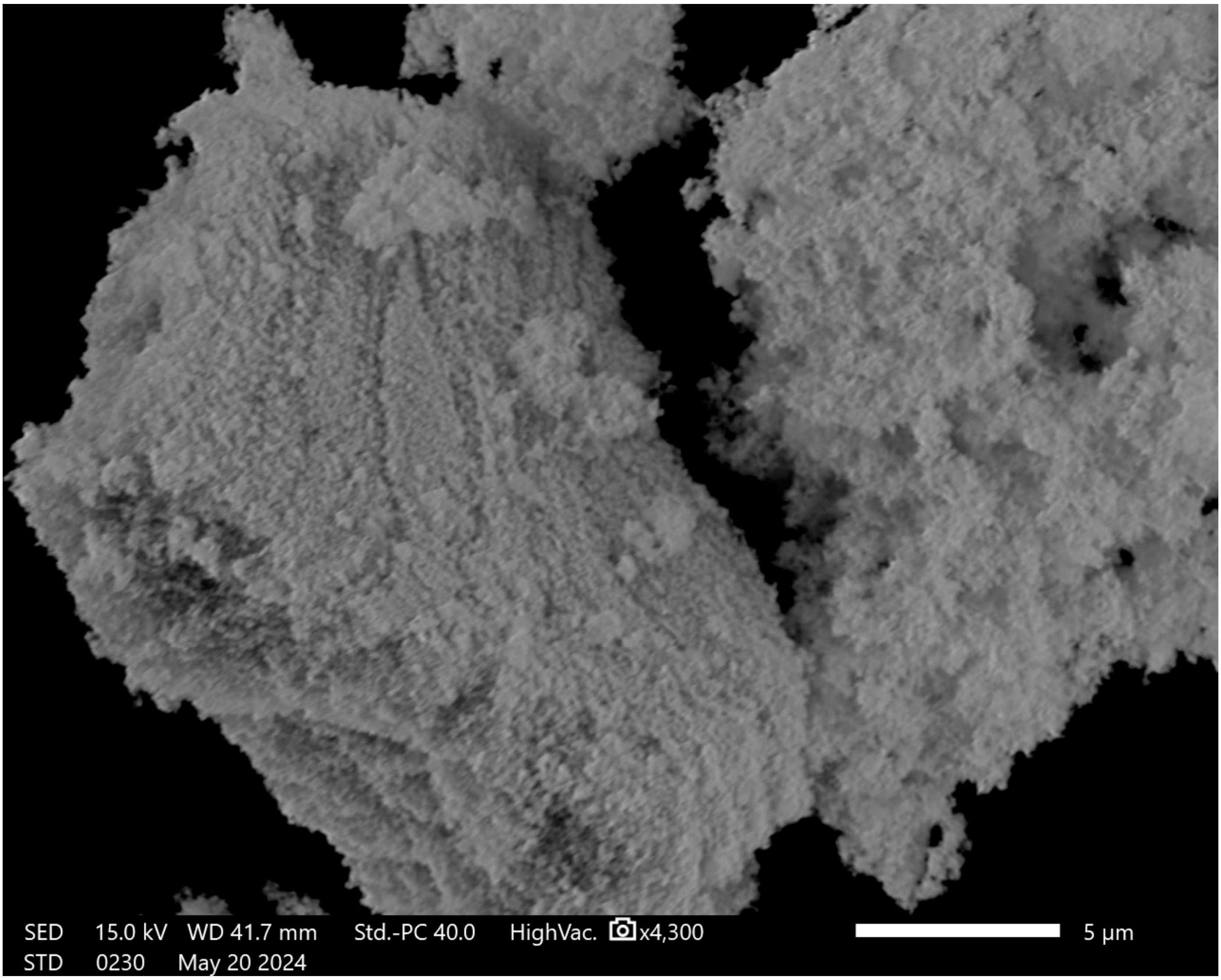

Figure S2. SEM captured photo of biosynthesized $ZnFe_2O_4$ NPs with sample to lens distance of ~42mm.

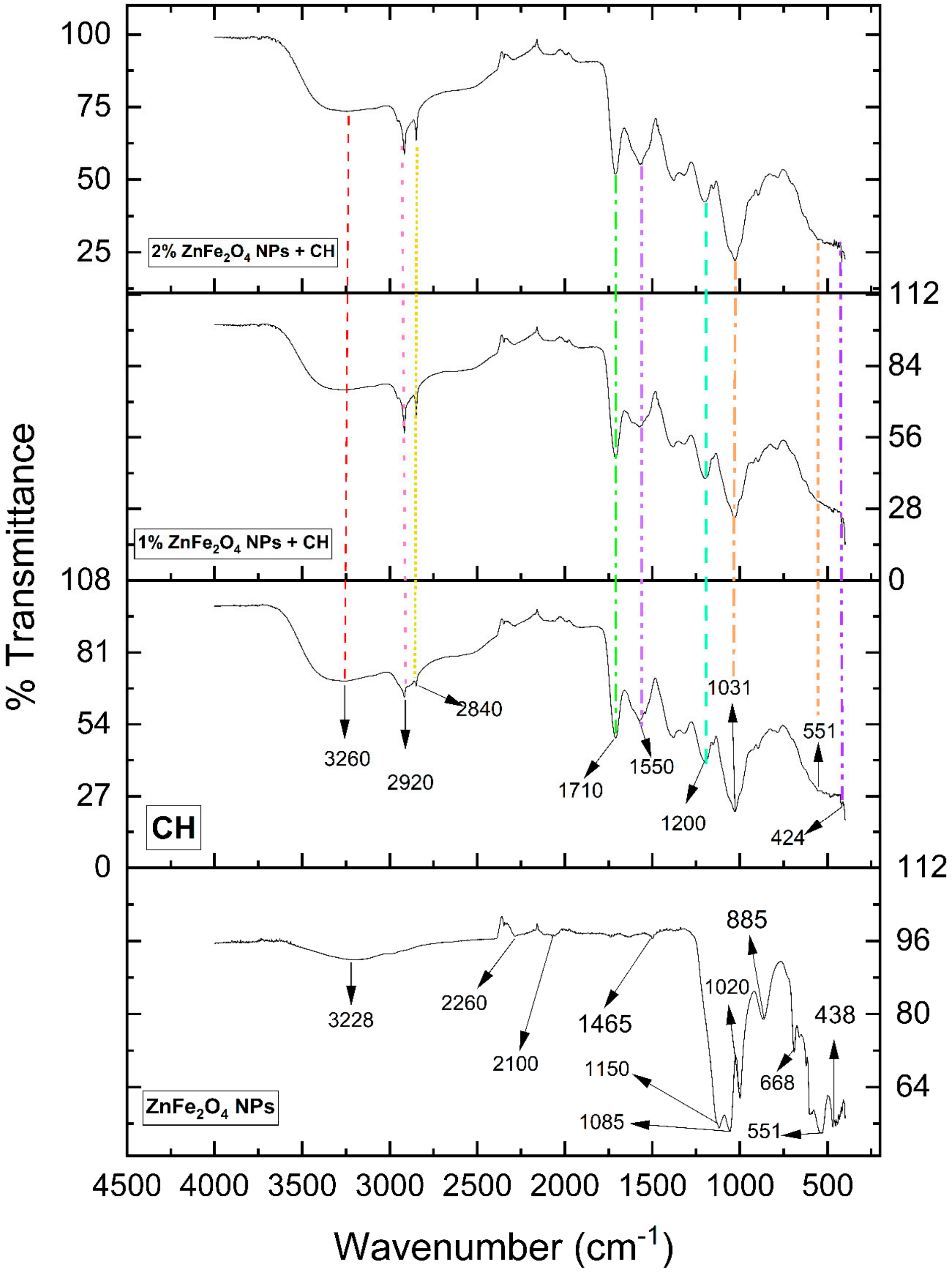

Figure S3. Plotted FTIR spectra for $ZnFe_2O_4$ NPs, Ch, and 1% and 2% $ZnFe_2O_4$ NPs containing CH films.

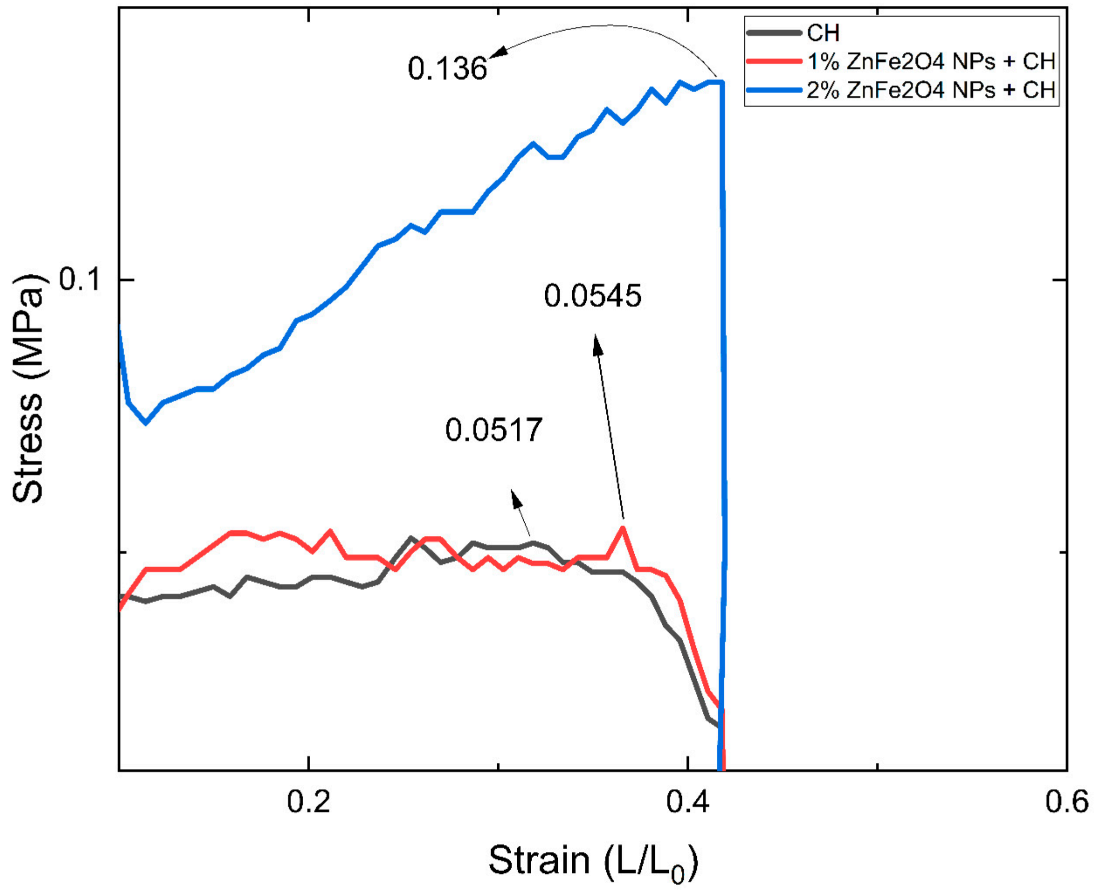

Figure S4. Puncture strength test plot for CH, 1% and 2% $ZnFe_2O_s$ CH films.

Tables:

Table S1. Crystaline size calculation of biosynthesized $ZnFe_2O_4$ NPs using Scherrer equation.

**hkl**	**Peak Position (2θ)**	**FWHM**	**Size (nm)**
111	18.041	0.479	17.53
220	30.331	0.282	30.46
311	36.832	1.218	7.17
400	43.634	1.084	8.24
422	54.574	0.482	19.36
511	57.871	0.701	13.52
400	63.459	0.727	13.41
**Average Crystalline Size**			**15.67**

Table S2. FTIR spectrum peaks and their correspondence.

FTIR Peak Position	Correspondence	FTIR Peak Position	Correspondence
424 – NPs Films	Zn – O vibration	1465 – $ZnFe_2O_4$ NPs	C – H bending
438 – $ZnFe_2O_4$ NPs	Zn – O vibration	1550 – CH + NPs Films	N – O stretching
551 – NPs Films + NPs	Fe – O vibration	1710 – CH + NPs Films	C = O stretching
668 – $ZnFe_2O_4$ NPs	C = C bending	2100 – $ZnFe_2O_4$ NPs	C ≡ C stretching
885 – $ZnFe_2O_4$ NPs	C = C bending	2260 – $ZnFe_2O_4$ NPs	C ≡ C stretching
1020 – $ZnFe_2O_4$ NPs	C – O stretching	2840 – CH + NPs Films	C – H stretching
1031 – CH + NPs Films	C – O stretching	2920 – CH + NPs Films	O – H stretching
1085 – $ZnFe_2O_4$ NPs	C – O stretching	3228 – CH + NPs Films	O – H stretching
1150 – $ZnFe_2O_4$ NPs	C – O stretching	3260 – $ZnFe_2O_4$ NPs	O – H stretching
1200 – CH + NPs Films	C – O stretching		

Table S3. %MC, %WS, %DS, TS, YM, %E and puncture strength values for bare CH films, 1% and 2% $ZnFe_2O_4$ NPs containing CH films

Sample	%MC	%WS	%DS	YM (MPa)	%E	TS (MPa)	Puncture test (MPa)
**CH**	7.228 ± 0.308	37.662 ± 1.098	9.740 ± 0.490	1.346	39.843	0.641	0.051
**1%**	8.334 ± 0.293	54.639 ± 2.498	13.526 ± 0.513	1.896	32.678	0.717	0.053
**2**	10.619 ± 0.388	61.352 ± 1.979	17.247 ± 0.839	2.007	29.853	0.835	0.136
